# Evaluation of Endothelial Dysfunction and Autophagy in Fibromyalgia-Related Vascular and Cerebral Cortical Changes and the Ameliorative Effect of Fisetin

**DOI:** 10.3390/cells11010048

**Published:** 2021-12-24

**Authors:** Fatma Mohamed Ghoneim, Salwa Mohamed Abo-Elkhair, Ayman Zaky Elsamanoudy, Dalia A. Shabaan

**Affiliations:** 1 Histology and Cell Biology Department, Faculty of Medicine, Mansoura University, Mansoura 35516, Egypt; fatmaghonaim@gmail.com (F.M.G.); dodosands@yahoo.com (D.A.S.); 2 Medical Biochemistry and Molecular Biology Department, Faculty of Medicine, Mansoura University, Mansoura 35516, Egypt; dr.salwakhair@gmail.com; 3 Clinical Biochemistry Department, Faculty of Medicine, King Abdulaziz University, Jeddah 21465, Saudi Arabia

**Keywords:** fibromyalgia, reserpine, fisetin, endothelial dysfunction, autophagy

## Abstract

Fibromyalgia (FM) is a common chronic pain syndrome that affects 1% to 5% of the population. We aimed to investigate the role of endothelial dysfunction and autophagy in fibromyalgia-related vascular and cerebral cortical changes in a reserpine-induced rat model of fibromyalgia at the histological and molecular levels and to study the ameliorative effect of fisetin. Forty adult female albino rats were divided into four groups (10 each): two control groups, the reserpine-induced fibromyalgia group, and the fisetin-treated group. The carotid arteries and brains of the animals were dissected. Frozen tissue samples were used for total RNA extraction and qPCR analysis of eNOS, caspase-3, Bcl-2, LC-3, BECN-1, CHOP, and TNF-α expression. Histological, immunohistochemical (eNOS), and ultrastructure studies were conducted. The carotid arteries revealed excessive autophagy and endothelial, vascular, and apoptotic changes. The cerebral cortex showed similar findings apart from endoplasmic reticulum stress. Additionally, there was decreased gene expression of eNOS and Bcl-2 and increased expression of caspase-3, LC-3, BECN-1, CHOP, and TNF-α. In the fisetin-treated rats, improvements in the histological and molecular results were detected. In conclusion, oxidative stress, enhanced apoptosis, and excessive autophagy are fundamental pathophysiologic mechanisms of reserpine-induced fibromyalgia. Moreover, fisetin has an ameliorative effect against fibromyalgia.

## 1. Introduction

Fibromyalgia (FM) is a common and complex chronic pain syndrome that affects 1% to 5% of the population [1]. The most common characteristic of fibromyalgia is chronic and prevalent pain without any apparent organic lesion that lasts for more than three months. The notable symptoms of FM include joint stiffness, sleep disruption, fatigue, cognitive impairment, and depression [2,3]. However, there are inconsistencies in the pathogenesis of FM, and the source of sensory inputs is unknown [4]; however, some hypotheses on peripheral and central pathophysiological mechanisms have been suggested. 

Endothelial dysfunction (ED) is a well-established risk factor that plays a pathophysiologic role in the development of atherosclerosis and in related disorders. ED describes a change in the endothelial cells that causes a decreased vasodilative response along with the reduced availability of nitric oxide as well as of other endothelium relaxing mediators. ED has also been shown to develop in different conditions, such as diabetes mellitus, arterial hypertension, chronic heart failure, and stroke [5]. It has previously been proposed that ED participates in the pathophysiology of fibromyalgia and fibromyalgia-related disorders [6]. 

Autophagy refers to a method of cellular recycling that encourages the competence of energy through the generation of ATP, monitors damage through eliminating organelles and nonfunctioning proteins, and regulates the degeneration of cytosolic elements via the autophagosomes [7]. It is the key catabolic pathway by which the organelles in the eukaryotic cells can be degenerated and recycled. Moreover, it is activated as a result of disorders that are related to environmental stress and pathologic situations [8]. Disorders affecting selective autophagy, oxidative stress, and its consequent mitochondrial dysfunctions are proposed to be some of the major players in the pathogenesis of fibromyalgia-related disorders [9].

As far as flavonoids are concerned, they are polyphenolic compounds that are characterized by antioxidant, anti-inflammatory, and antinociceptive effects [10]. Fisetin (7,3’,4’-flavon-3-ol) is a dietary plant polyphenol flavonoid that possesses anti-inflammatory, antioxidant, neuroprotective, anticarcinogenic, cardioprotective, antiallergic, and antiulcer properties [11]. Furthermore, it is used for the protection and treatment of certain neurodegenerative diseases such as as Parkinson’s disease and Alzheimer’s disease [11,12]. Although fisetin’s potential protective use was studied in an experimental model of fibromyalgia by Yao et al. [12], its protective mechanism still needs further attention and justification. 

As such, the present study was performed to investigate the pathophysiologic role of endothelial dysfunction and autophagy in fibromyalgia-related vascular and cerebral cortical changes in a reserpine-induced rat model of fibromyalgia at the histological and molecular levels as well as to study the potential ameliorative effect of fisetin.

## 2. Materials and Methods

The current study is an experimental animal study that was conducted in the Histology and Cell Biology and Medical Biochemistry and Molecular Biology departments at the Faculty of Medicine of Mansoura University, Egypt.

### 2.1. Drugs and Animals

Reserpine was obtained from Sigma Chemical Co., St. Louis, MO, USA, and was diluted in 0.5% glacial acetic acid and distilled water (vehicle) before administration.

Fisetin (≥98%) was purchased from Sigma-Aldrich, Merck KGaA, and was dissolved in 1% DMSO.

The FM induction protocol of our study was conducted and initiated by obtaining a total of 46 adult female albino Sprague Dawley rats weighing 200–250 g and that were aged 8–10 weeks old from the experimental animal house in the Faculty of Science, Mansoura University, Egypt. The female rats were kept inside polypropylene cages (5 animals per cage) that were maintained at 22 ± 1 °C with an average humidity level of 40–50% and a 12 h light/dark cycle. Two weeks earlier, they had been accommodated in a quiet non-stressful atmosphere as an acclimation period and had free access to standard pellet chow and water throughout the experiments. 

### 2.2. Induction of Fibromyalgia

A rat model of fibromyalgia was induced in 26 rats through the administration of reserpine subcutaneously into the back of rats at various injection sites on the flank at a dose of 1 mg/kg body weight daily for three consecutive days [13]. To ensure the verification of the development of the fibromyalgia model, the following behavioral tests were conducted: Randall–Selitto paw pressure test, an index of mechano-hyperalgesia [14], the Hargreaves test to assess thermal pain sensation in rodents, and the tail suspension test (TST), which showed the alternate periods of agitation and immobility as the rat was suspended by the tail [12] (Table 1). After developing and verifying the fibromyalgia rat model, ten rats were randomly selected for each of the two fibromyalgia groups after excluding those who did not develop fibromyalgia.

Consequently, in the current study, 40 adult female albino Sprague Dawley rats were separated into four groups (10 rats per group) as follows:

Group I (negative control group): Rats were injected subcutaneously with distilled water and 0.5% acetic acid for three successive days and then received 1% DMSO solution for the next three weeks.

Group II (positive control group): Rats were injected subcutaneously with distilled water and 0.5% acetic acid for three successive days and then received fisetin (25 mg/kg) orally for the next three weeks [11]. 

Group III (reserpine-treated group): Rats were injected subcutaneously with reserpine as previously described and were then treated orally with the vehicle for the next three weeks [13].

Group IV (reserpine and fisetin-treated group): Tats were injected subcutaneously with reserpine as previously described and were then treated orally with fisetin (25 mg/kg) for the next three weeks [12]. 

### 2.3. Sampling 

Once all of the experiments were completed and after 12 h of overnight fasting, each rat was anesthetized with diethyl ether. A blood sample was then withdrawn from the retro-orbital venous plexus under a complete aseptic condition through the use a one-use plastic syringe. Sera were prepared by centrifugation at 5000 rpm for 15 min and were stored at −80 °C until the biochemical investigations were performed.

The heart was exposed via a thoracic incision for histological and molecular studies. Intra-cardiac perfusion was conducted using normal saline followed by 10% neutral-buffered formalin for the partial fixation of the specimens. The carotids arteries and brains were exposed and were carefully dissected. For the molecular investigations, about 20–40 mg tissue samples from the cerebral cortex and carotid artery were snap-frozen in liquid nitrogen and were stored at −80 °C. For total RNA extraction and for the qPCR analysis of eNOS, caspase-3, Bcl-2, LC-3, BECN-1, CHOP, and TNF-α expression, frozen tissue samples were utilized.

### 2.4. Histological Study

The histological study was initiated by fixing the tissue samples in 10% buffered formalin, and the samples were then dehydrated, and a paraffin block was prepared. Paraffin sections (5 μm thick) were prepared and were stained with hematoxylin and eosin (H&E) and Masson’s trichrome [15].

With respect to transmission electron microscopy, 2.5% glutaraldehyde buffered with 0.1 M Sodium Cacodylat (pH 7.2) was used to fix the tissue samples for 2 hours, and the samples were then washed with the same buffer three times. After washing, an additional glutaraldehyde-osmium tetroxide double immersion fixation was applied, and the samples were then dehydrated in an increasing series of ethanol. After being dehydrated, the samples were immersed in propylene oxide and were then embedded in an epoxy resin mixture. After that, 1 um thick sections were obtained to be stained with 1% toluidine. LKB ultramicrotome was used to obtain ultrathin sections (80–90 nm) that were stained with uranyl acetate and lead citrate [16]. The last step was to examine the sections using a transmission electron microscope (JEOL JEM-2100) (Gatan Inc., Tokyo, Japan) in the Electron Microscopic Unit, Faculty of Agriculture, Mansoura University, Egypt.

For the immunohistochemical study, paraffin sections of the brain and carotid arteries were mounted on positive slides and were immunostained using the avidin–biotin technique [17]. The sections were deparaffinized, rehydrated, and washed in PBS. Then, they were embedded in 0.01% H_2_O_2_ to suppress endogenous peroxidase activity. The sections were boiled in citrate buffer (pH 6) for 10 min and were then cooled and washed in PBS for antigen retrieval. In order to decrease non-specific binding, the sections were incubated with 10% normal goat serum for 30 min followed by incubation for 30 minutes with 1:100 dilutions of primary eNOS rabbit polyclonal antibody IgG (ab 5589, Abcam, Cambridge, UK). Then, the sections were treated with biotinylated secondary antibody (Dako-K0690; Dako Universal LSAB Kit) and streptavidin–biotin peroxidase complex (Dako-K0690) for 30 min. The immunoreactivity was visualized using 3,3′diaminobenzidine (DAB)-hydrogen peroxide as a chromogen (Sigma-D5905; Sigma– Aldrich Company Ltd., Gillingham, UK) for 5 min. The counterstain that was Mayer’s hematoxylin. In the end the sections were dehydrated, cleared, and mounted in DPX (Sigma). Negative control sections were obtained after replacing the 1ry antibodies with phosphate-buffered saline.

### 2.5. Morphometric Study

The morphometric study was conducted by examining ten non-overlapping fields at a magnification of (×400) from each rat (5 rats/group) by using Leica Qwin 500 image analysis software on an IBM-operated computer system (Leica Microsystems, Wetzlar, Germany) to measure:

1. The carotid intima-media thickness (IMT) from the innermost endothelial boundary to the outermost tunica media, which is difficult to differentiate between the intima and media.
2. The mean area percentage of collagen was measured inside a standard measuring frame of a known area: the blue area was masked by the collagen fibers and was then measured and expressed as an area percent to the area of the standard measuring frame.
3. The mean area percentage of immune reaction for eNOS.

The mitochondrial number was measured by examining ten non-overlapping fields that were obtained by EM at a magnification of (×8200) from each rat (5 rats/group) [18].

### 2.6. Molecular Study

Real-Time qPCR (RT-qPCR): Total RNA from rat carotid artery and cerebral cortex tissue samples (~25 mg) were extracted using Trizol Reagent (Invitrogen, Waltham, MA, USA) according to the manufacturer’s instructions and were stored at −80 °C. The concentration and purity of total isolated RNA were determined by Nanodrop spectrophotometry. The reverse transcription reaction for cDNA synthesis was performed with ~200 ng total RNA using a Maxima First Strand cDNA Synthesis Kit (Thermo Scientific, Waltham, MA, USA, cat No. #K1641). The rat carotid artery and cerebral cortex mRNA expressions of eNOS, caspase-3, Bcl-2, LC3, and BECN-1 were quantified by real-time PCR using the Applied Biosystem 7500, real-time PCR detection system (Life Technology, Carlsbad, CA, USA) with “HERAPLUS SYBR® Green qPCR Master Mix” (2X) (Willowfort, Birmingham, UK, cat. No. WF10308001). Reaction mixtures were incubated for 10 min at 95 °C, followed by 40 cycles of 15 s at 95 °C and 30 s at 60 °C. The primer sequences used were rat eNOS: forward, 5′-ACCGCCACACAGTAAATCCA-3′; re-verse,5′-TGCCAACAGGAAGCTGAGAG-3′[19], rat caspa-se-3: forward, 5′-GTGGAACTGACGATGATATGGC-3′; reverse, 5′-CGCAAAGTGACTG-GATGAACC-3′ [20], rat Bcl-2: forward, 5′-TGTGGATGACTGACTACCTGAACC-3′; re-verse 5′- CAGCCAGGAGAAATCAAACAGAGG-3′) [20], rat LC3: forward, 5′-CCTGCTGCTGGCCGTAGT-3′; reverse, 5′- TGATGAAGTCTTCCTGCCAAAA-3′ [21], rat BECN-1: forward, 5′-AGCACGCCATGTATAGCAAAGA-3′; reverse, 5′-GGAA-GAGGGAAAGGACAGCAT-3‘[21],rat CHOP: forward, 5′-GAAAGCAGAAACCGGTCCAAT-3′; re-verse 5′-GGATGAGATATAGGTGCCCCC-3′ (GenBank: U36994.1). Rat TNF-α: forward, 5′-AAT GGC CTC CCT CAT CAG TT-3′; reverse, 5′-CCA CTT GGT GGT TTG CTA CGA-3′ [22]. The primers sequences for rat β-actin were 5’-AA-GATCCTGACCGAGCGTGG-3’ (Forward) and 5’-CAGCACTGTGTTGGCATAGAGG-3’ (Reverse) [20]. The expression of the analyzed genes was normalized to that of the internal control gene, the β-actin, using the comparative ΔΔCT method.

### 2.7. Biochemical Investigations

Oxidative stress markers were measured in the sera of all of the rats. The lipid peroxidation product, malondialdehyde (MDA), was estimated based on the thiobarbituric acid reaction [23], and the results were expressed as μmol MDA/L. The total antioxidant capacity (TAC) was estimated by the commercially available colorimetric kits (Cat.No. # TA 25 12, Giza, Egypt) supplied by Bio-Diagnostics, Dokki, Giza, Egypt [24], and the results were expressed as mmol/L. The serum nitric oxide (NO) was measured by the colorimetric method (Nitric Oxide Assay Kit, Abcam Co., Boston, MA, USA, ab272517), and the results were expressed as *μ*mol/L.

### 2.8. Statistical Analysis

Comparisons were performed by analysis of variance (one-way ANOVA test) followed by Tukey’s test (post hoc tests) using the social sciences statistical package (SPSS 27.0, IBM/SPSS Inc., Chicago, IL, USA). The data that were obtained from behavioral tests, biochemical, molecular, and morphometric studies were expressed as the mean ± standard deviation. Differences were considered to be statistically significant when *p* < 0.05. 

## 3. Results

### 3.1. Histological Results

#### 3.1.1. Carotid Artery

##### Light Microscopic Results

**Figure 1:**
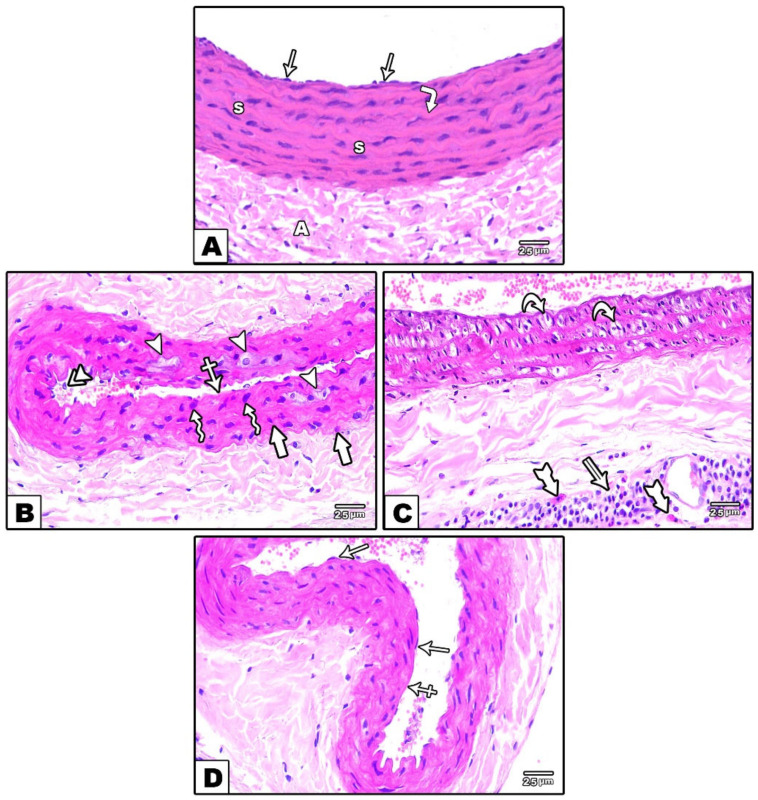
Photomicrographs of sections in the carotid artery stained with H&E. (**A**): In the control groups, the wall of the carotid artery consisted of thin tunica intima lined with endothelial cells (arrows), the intima mingles with the underlying media, which is formed from smooth muscle fibers (S), and wavy elastic fibers (corner arrow). The adventitia (A) is composed of loose connective tissue. (**B,C**): Reserpine-treated group. (**B**): Loss of some endothelial cells (crossed arrow) and the shedding of others (double arrowheads) in the lumen. Tunica media contains foam cells (arrowhead) and smooth muscle fibers with plump nuclei (zigzag arrows) or dark pyknotic nuclei (thick arrow). (**C**): Tunica media shows cytoplasmic vacuolation in the smooth muscle fibers (curved arrows). Tunica adventitia appears thick with inflammatory cell infiltration (double arrow) and mast cells (tailed arrows). (**D**): The fisetin-treated group **demonstrated** marked improvement of the wall and endothelial lining (arrows), apart from the loss of few epithelial cells (crossed arrow).

A microscopic examination of the hematoxylin and eosin-stained sections of the carotid arteries of control groups (groups I and II) were similar and exhibited the well-known typical structure of the carotid artery. Its walls were formed by three layers: the tunica intima, tunica media, and tunica adventitia. Regarding the tunica intima, it was thin, lined with endothelial cells, and mingled with the underlying media, which was formed from smooth muscle fibers with large oval nuclei and wavy elastic fibers. The tunica adventitia was composed of loose connective tissue (Figure 1A). In group III (Reserpine treated group), some endothelial cells in the tunica intima were lost, and others were exfoliated in the lumen. There were degenerative changes in the form of foam cells in the tunica media, some smooth muscle cells appeared with plump nuclei, and others appeared with dark pyknotic nuclei (Figure 1B). There was vacuolation in the cytoplasm of some of the smooth muscle cells in the tunica media. The tunica adventitia appeared to be thick and demonstrated the presence of a mixed inflammatory cell infiltrate and mast cells (Figure 1C). Treatment with fisetin (group IV) resulted in a marked improvement of the structure of the carotid artery compared to the untreated group, apart from the loss of a few endothelial cells from the tunica intima (Figure 1D). 

**Figure 2:**
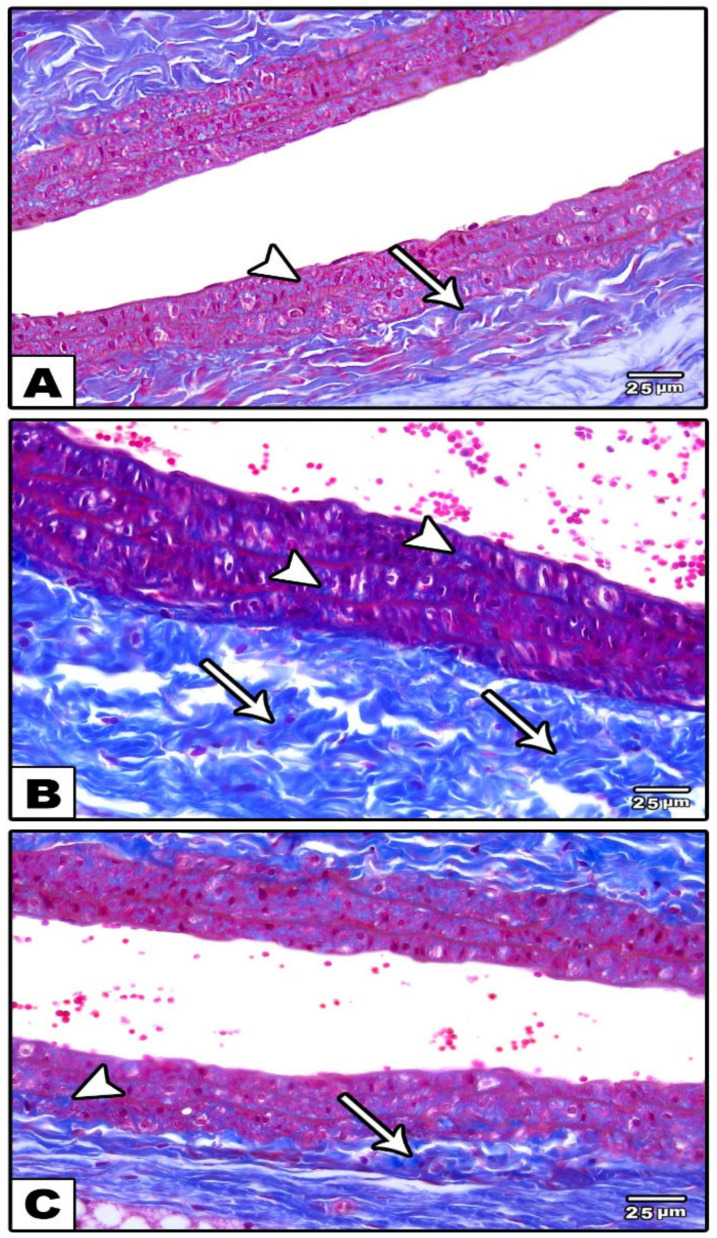
Photomicrographs of sections in the carotid artery stained with Masson’s trichrome stain. (**A**): In the control groups, minimal collagen fibers in tunica media (arrow) and in the tunica adventitia (arrowhead) are observed. (**B**): In the reserpine-treated group, a massive deposition of collagen fibers in tunica media (arrows) and in the tunica adventitia (arrowheads) is observed. (**C**): In the fisetin-treated group, moderate amounts of collagen fibers in tunica media (arrow) and in the tunica adventitia are observed(arrowhead).

In the Masson’s trichrome-stained sections, minimal collagen fibers were detected in the tunicae media and in the adventitia of carotid arteries of control groups (groups I and II) (Figure 2A), while in group III (reserpine-treated group), there was a massive deposition of collagen fibers (Figure 2B). In group IV (fisetin-treated group), moderate amounts of collagen fibers were detected in the tunicae media and in the adventitia (Figure 2C).

**Figure 3:**
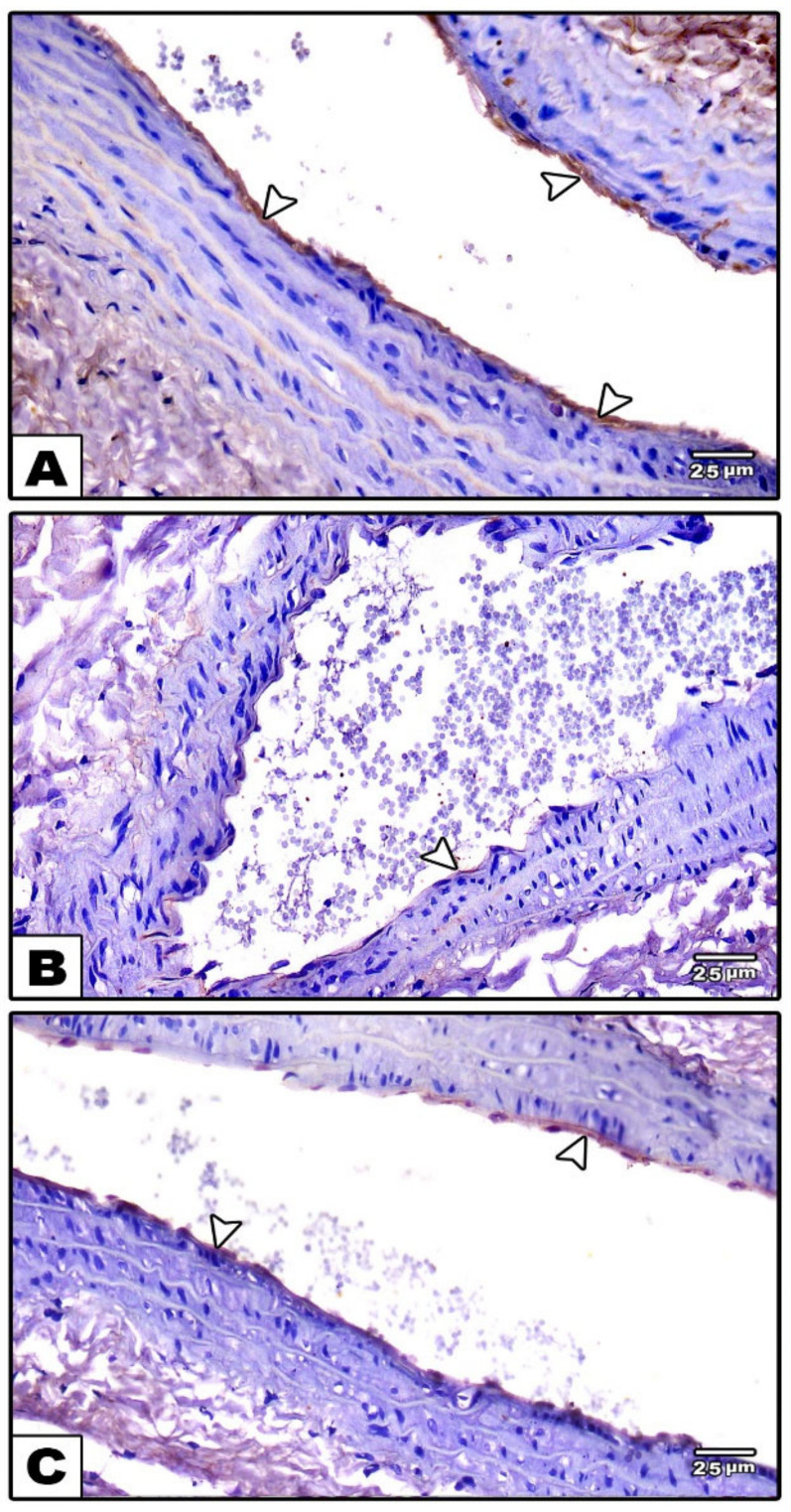
Photomicrographs of sections in the carotid artery immunohistochemically stained with eNOS. (**A**): In the control groups, strong positive eNOS immunoreactivity is observed in the endothelial lining of the tunica intima (arrowheads). (**B**): In the reserpine-treated group, decreased eNOS immunoreactivity (arrowheads) is observed (**C**): In the fisetin-treated group, weak positive eNOS immunoreactivity (arrowheads) in the endothelial lining of the tunica intima is observed.

Labeling the segments of the carotid arteries from control rats (groups I and II) with the monoclonal antibody for eNOS revealed a high level of its expression in the endothelial cell lining of the tunica intima (Figure 3A), whereas the carotid arteries from the reserpine-treated rats (group III) showed a week-level of eNOS expression compared to those from the control groups (Figure 3B). In group IV (fisetin-treated group), the eNOS expression in the endothelial cell lining of the tunica intima was increased compared to in the reserpine-treated group (Figure 3C)

##### Electron Microscopic Results

**Figure 4:**
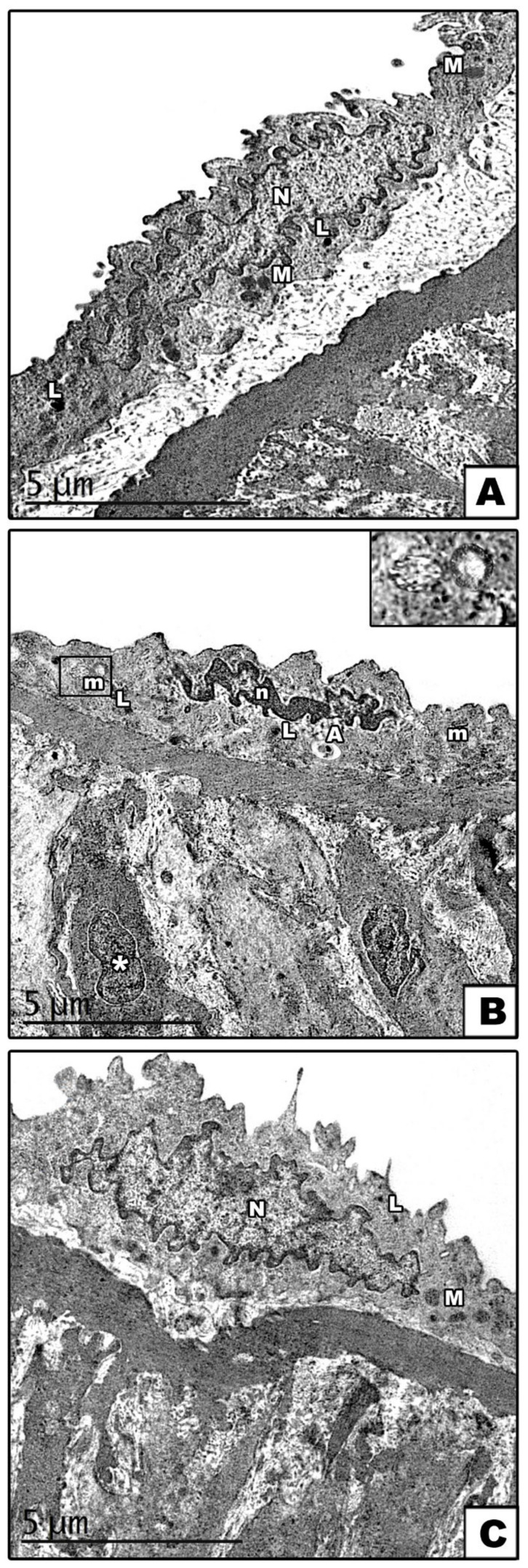
Electron micrographs of sections in the carotid arteries (**A**): In the control groups, the endothelial cells have a euchromatic nucleus (N), and its cytoplasm contains mitochondria (M) and few lysosomes (L). (**B**): In the reserpine-treated group, small heterochromatic nuclei (n) in the endothelial cells, mitochondria (m) with destructed cristae, numerous lysosomes (L), and autophagosome (A) are observed in the cytoplasm. Note the presence of apoptotic smooth muscle cells (asterisks) with a heterochromatic nucleus and condensed cytoplasm in the tunica media. The boxes indicate the destructed cristae of the mitochondria. (**C**): In the fisetin-treated group, normal endothelial cells with a euchromatic nucleus (N), normal mitochondria (M), and few lysosomes (L) are observed.

An examination of the ultrathin sections of the carotid arteries from the control groups (groups I and II) revealed that the arteries demonstrated a normal endothelial surface. The nuclei of the endothelial cells were euchromatic, and the cytoplasm contained mitochondria with normal cristae and a few lysosomes (Figure 4A). In group III (reserpine-treated group), the nuclei of the endothelial cells were small and heterochromatic. The cytoplasm contained mitochondria with destructed cristae, numerous lysosomes, autophagosomes, tunica media, and apoptotic smooth muscle cells with a small heterochromatic nucleus in condensed cytoplasm (Figure 4B). In the fisetin-treated group, an apparently normal endothelial cell ultrastructure was observed, with a euchromatic nucleus, normal mitochondria, and few lysosomes (Figure 4C).

#### 3.1.2. Cerebral Cortex

##### Light Microscopic Results

**Figure 5:**
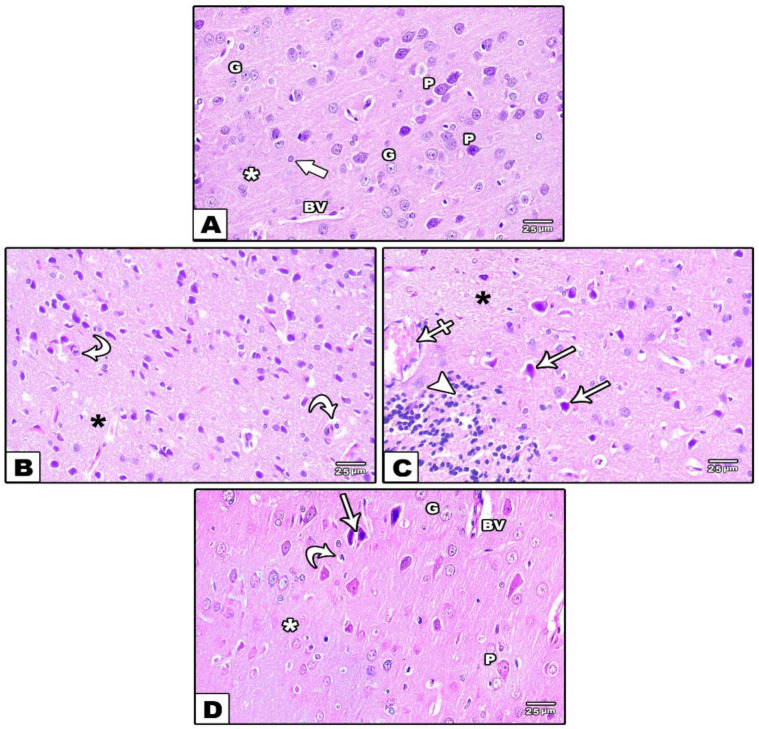
Photomicrographs of sections in the cerebral cortex stained with H& E. (**A**): In the control groups, large pyramidal cells and scattered granular ones can be observed. The pyramidal cells (P) have long apical dendrites, basophilic cytoplasm, and large vesicular nuclei. The granular cells (G) have rounded cell bodies with large rounded vesicular nuclei. The neuropil (white asterisk) contains the nuclei of neuroglial cells (thick arrow) and normal blood vessels (BV). (**B**,**C**): In the reserpine-treated group, (**B**) apoptotic cells (curved arrows) with small darkly stained nuclei and little acidophilic cytoplasm with vacuolation (black asterisk) in the neuropil are observed. (**C**): Perineuronal empty spaces (arrows) around deformed and shrunken pyramidal cells are visible. Vacuolation (black asterisk), inflammatory cellular infiltration (arrowhead), and dilated congested blood vessels (crossed arrow) are noticed in the neuropil. (**D**): In the fisetin-treated group, normal pyramidal (P) and granular (G) cells are seen. Few shrunken cells (arrows) with dark nuclei and perineuronal empty spaces and few apoptotic cells (curved arrow) are noticed. Normal neuropil (white asterisk) with non-congested blood vessels (BV) is seen.

An examination of the hematoxylin and eosin-stained sections of the cerebral cortexes from the control groups (groups I and II) brough the normal histological architecture of the pyramidal and granular cells in the cerebral cortical layers to light. The pyramidal cells had long apical dendrites, basophilic cytoplasm, and large vesicular nuclei. The granular cells had rounded cell bodies and large rounded vesicular nuclei. The homogenous eosinophilic background (neuropil) between the nerve cells contained the nuclei of neuroglial cells and normal blood vessels (Figure 5A). In group III (reserpine-treated group), marked morphological changes were visualized; the cerebral cortex exhibited many apoptotic cells with small darkly stained nuclei and little acidophilic cytoplasm. Perineuronal empty spaces appeared around deformed and shrunken pyramidal cells. What is more, the neuropil showed vacuolation, inflammatory cellular infiltration, and dilated congested blood vessels (Figure 5B,C). In group IV (fisetin-treated group), there was a noticeable improvement in the structure of the cerebral cortex, apart from a few shrunken pyramidal cells with dark nuclei surrounded by empty spaces and few apoptotic cells (Figure 5D).

**Figure 6:**
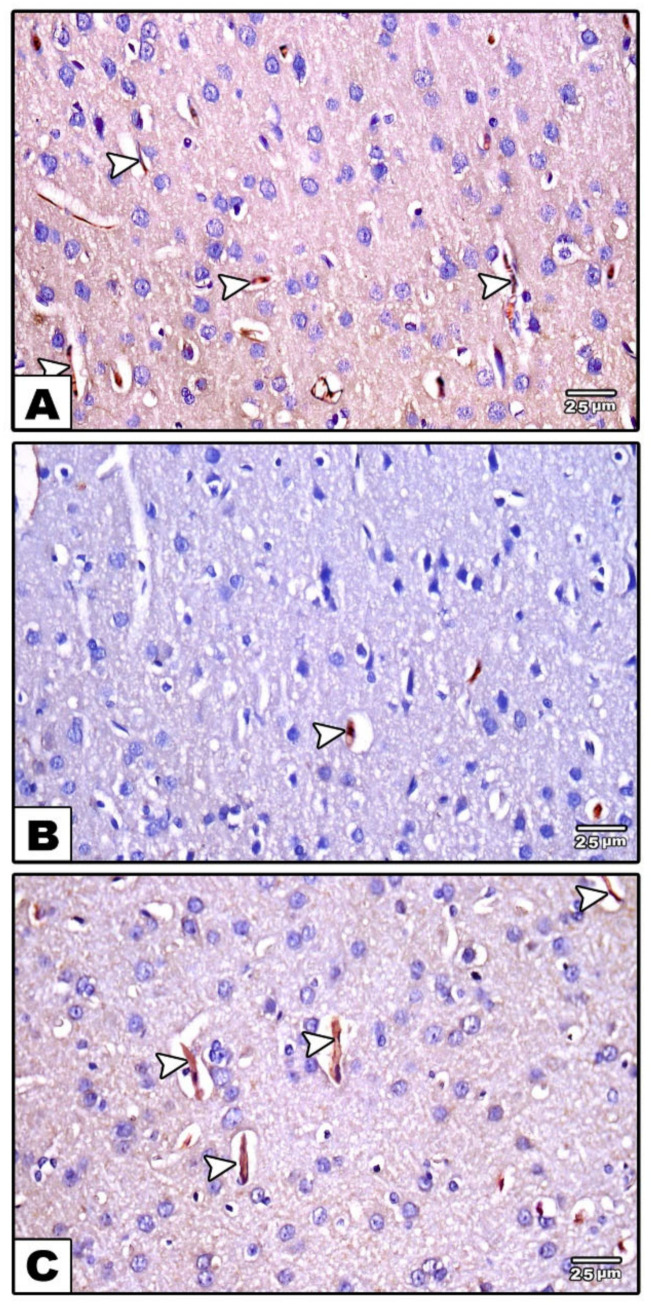
Photomicrographs of sections in the cerebral cortex immunohistochemically stained with eNOS. (**A**): In the control group, strong positive eNOS immunoreactivity is seen in the endothelial lining of the blood vessels (arrows) (**B**): In the reserpine-treated group, week positive eNOS immunoreactivity (arrows) can be observed (**C**): In the fisetin-treated group, mild positive eNOS immunoreactivity in the endothelial lining of blood vessels (arrows) can be seen.

In the immunohistochemically stained sections of the control groups (groups I and II), eNOS was strongly expressed in the endothelial lining of the blood vessels found in the neurophil (Figure 6A). Compared to the untreated control groups, its expression was decreased in group III (reserpine-treated group) (Figure 6B), while it was increased after treatment in the fisetin-treated group (group IV) (Figure 6C).

###### Electron Microscopic Results

**Figure 7:**
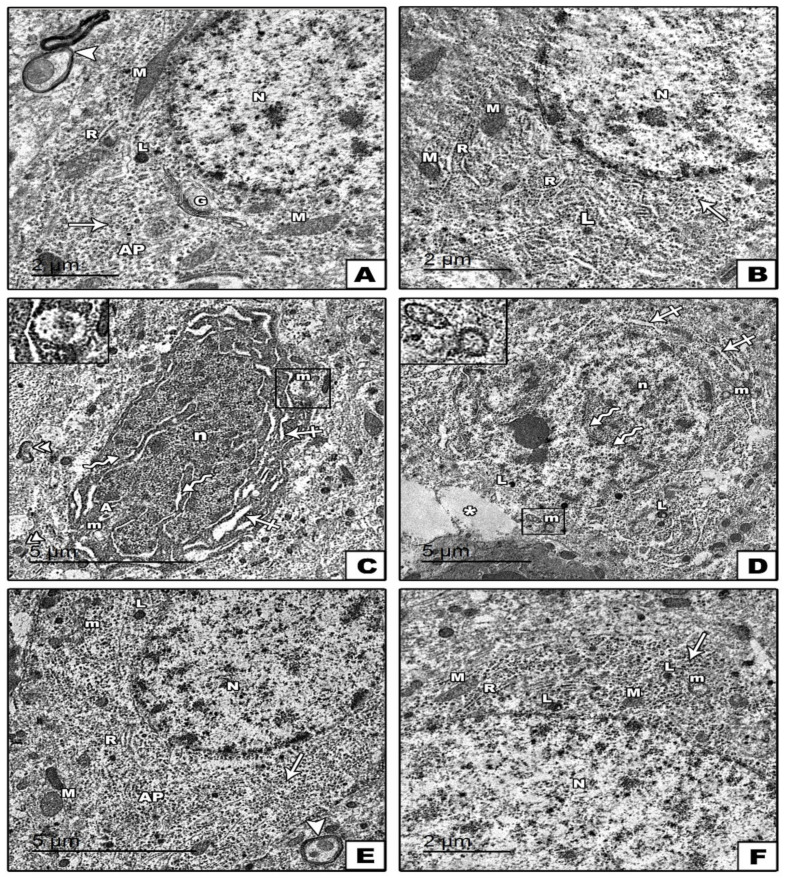
Electron micrographs of sections in the cerebral cortex. (**A**,**B**): In the control groups, (**A**) pyramidal cells with long apical dendrites (AP) and rounded euchromatic nuclei (N) are observed. The cytoplasm shows rER cisternae (R), polyribosomes (arrows), few lysosomes (L), mitochondria (M) with normal cristae pattern, and Golgi saccules (G). Notice the presence of myelinated nerve fibers with the regular smooth contour of its myelin sheath (arrowhead) in the surrounding neuropil (**B**): A granular cell with a rounded euchromatic nucleus (N) and contains rER cisternae (R), polyribosomes (arrows), few lysosomes (L), and mitochondria (M) with a normal cristae pattern in the cytoplasm. (**C**,**D**): In the reserpine-treated group, (**C**) pyramidal cells show irregular heterochromatic nuclei (n) with corrugated nuclear membranes surrounded by a wide perinuclear space (zigzag arrows). Mitochondria with destructed cristae (m) autophagosome (A) and markedly dilated rER cisternae (crossed arrows) are seen in the cytoplasm. The nerve fibers (double arrowheads) in the surrounding neuropil have a disrupted myelin sheath. (**D**): The granular cell shows an irregular nucleus (n) with a corrugated nuclear membrane (zigzag arrows). The cytoplasm reveals dilated rER cisternae (crossed arrows), numerous lysosomes (L), and mitochondria (m) with destructed cristae. The boxes indicate the destructed cristae of mitochondria. (**E**,**F**): In the fisetin-treated group, (**E**) normal pyramidal cell with apical dendrites (AP) are observed. (**F**): Normal granular cell. In both figures (**E**,**F**), the nucleus (N) is rounded and euchromatic, and the cytoplasm contains average rER (R), mitochondria (M) with a normal cristae pattern, polyribosomes (arrows), and few lysosomes (L). Few mitochondria (m) with destructed cristae are noticed.

An examination of ultrathin sections of groups I and II (control groups) of the cerebral cortexes revealed the normal ultrastructure picture of the pyramidal and granular cells. The pyramidal cells appeared with long apical dendrite and euchromatic nuclei. The cytoplasm contained rER cisternae, scattered polyribosomes, few lysosomes, mitochondria with normal cristae pattern, and Golgi saccules. Myelinated nerve fibers with the regular smooth contour of its myelin sheath were also observed in the surrounding neuropil (Figure 7A). The granular cells had rounded euchromatic nuclei, and in the cytoplasm, there were mitochondria with a normal cristae pattern, few lysosomes, scattered polyribosomes, and rER cisternae (Figure 7B). In group III (reserpine-treated group), degenerative changes in the pyramidal and granular cells were observed. The pyramidal cells showed irregular heterochromatic nuclei with corrugated nuclear membranes surrounded by wide perinuclear spaces. The cytoplasm showed mitochondria with destructed cristae, autophagosomes, and markedly dilated rER cisternae. Nerve fibers with a disrupted myelin sheath were also seen in the surrounding neuropil (Figure 7C). The granular cells exhibited an irregular nucleus with a corrugated nuclear membrane, and its cytoplasm revealed mitochondria with destructed cristae, numerous lysosomes, and dilated rER cisternae (Figure 7D). In group IV (fisetin-treated group), a remarkable improvement was observed in the form of a regular contour in both the pyramidal and granular cells. The nuclei of both cells appeared rounded euchromatic. Their cytoplasm contained normal rER cisternae, mitochondria with a normal cristae pattern, polyribosomes, and a few lysosomes. However, few mitochondria with destructed cristae were still noticed (Figure 7E,F).

### 3.2. Biochemical Study Results

A biochemical study of oxidative stress biomarkers confirmed an endothelial dysfunction role in FM pathogenesis. The reserpine-induced fibromyalgia group showed a significant increase in serum MDA and NO levels, with a significant decrease in the total antioxidant capacity (TAC). These results were significantly reversed (even do not reach control group levels) by fisetin treatment (Table 1).

### 3.3. Morphometric, Molecular and Statistical Results

A non-significant change was inspected between the two control groups regarding the intima-media thickness of the carotid artery. A statistically significant increase was observed in the reserpine-treated group compared to in the control groups. A non-significant change in the reserpine + fisetin-treated group compared to the control groups and a significant decrease compared to the Reserpine treated group was also observed. Furthermore, there was a statistically significant increase in the area % of the collagen in the tunica media and in adventitia of the carotid arteries in the reserpine-treated group, and a non-significant change in the reserpine + fisetin-treated group when compared to the control groups was detected. Quantification of the number of the mitochondria revealed an increased mitochondrial number in the reserpine-treated group compared to in the control groups and a significant decrease in the reserpine + fisetin treated-group. A significant decrease in the area % of eNOS expression in the reserpine-treated group and a non-significant change in the reserpine + fisetin-treated group was noticed compared to the control groups. (Table 2).

Regarding the mitochondrial number in the cerebral cortex, we observed a statistically significant increase in the reserpine-treated group compared to in the control groups and a significant decrease in the reserpine + fisetin-treated group. In terms of the area percentage of eNOS expression, there was a non-significant change between the negative and positive control groups and a statistically significant decrease in the reserpine-treated group compared to in the control groups. A non-significant difference in the reserpine + fisetin-treated group was observed when compared to the control groups, and a significant increase was observed compared to in the reserpine-treated group (Table 3).

At the molecular level and in order to investigate the pathophysiologic role of endothelial dysfunction, apoptosis, and autophagy in fibromyalgia and the potential ameliorative effect of fisetin, the mRNA gene expression of eNOS, Bcl-2, caspase-3, LC-3, BECN-1, CHOP, and TNF-α were analyzed in both the carotid artery and in cerebral cortex. Inconsistent with the results of immunohistochemical expression in both the carotid artery and in the cerebral cortex, eNOS mRNA was significantly decreased in the reserpine-treated group, indicating the role of endothelial dysfunction in FM pathogenesis. Fisetin induced a significant improvement in eNOS gene expression (*p* < 0.001) (Table 2 and Table 3).

Reserpine-induced fibromyalgia was associated with stimulated apoptosis, as shown by the significant increase in caspase-3 expression and decreased Bcl-2 mRNA gene expression in the carotid artery and in the cerebral cortex. This apoptosis induction was significantly reduced by fisetin (*p* < 0.001). Moreover, an inflammatory reaction was indicated by increased TNF-α in both the carotid and cerebral tissues, and ER stress was indicated by the high expression level of CHOP gene expression, which was more evident in the cerebral cortical tissues than it was in the carotid vessels.

Moreover, autophagy was also induced in reserpine-induced fibromyalgia, as confirmed by the significant increase in the gene expression of the autophagy markers (LC-3, BECN-1) in the reserpine-treated group that was significantly ameliorated by fisetin treatment.

## 4. Discussion

In the present study, reserpine-induced fibromyalgia was selected to be our animal model. Reserpine induces the systemic depletion of the biogenic amines (dopamine, norepinephrine, and 5-hydroxytryptamine) in the spinal cord, thalamus, and prefrontal cortex [25]. This effect is associated with significant changes in the behavioral diagnostic tests of fibromyalgia [26], as observed in our study.

The etiopathogenesis of fibromyalgia is not fully understood and is still under debate [27]. The prominent role of inflammation in fibromyalgia with cytokine release disorders and its potential function in inducing and maintaining the chronic pain that is associated with fibromyalgia was studied by Kadetoff et al. [28]. In this study, inflammation was evidenced by the appearance of inflammatory cell infiltrate in the tunica adventitia along with higher TNF-α gene expression.

There is lower cerebral blood flow in FMS patients with a positive correlation between the emotional and cerebral functional variables and in the cerebral blood flow variability [29]. These findings propose an interconnected relationship between vascular and cerebral damage in the pathogenesis of FMS-related disorders. As a result, we aimed to investigate the pathophysiologic role of endothelial dysfunction and autophagy in fibromyalgia-related vascular and cerebral cortical changes.

In the present study, light microscopic examination of the carotid arteries revealed evident endothelial and vascular damage in the form of the loss of some endothelial cells and of exfoliation of others in the lumen of the carotid artery. Staining the carotid vascular wall by Masson’s trichrome stain revealed the massive deposition of collagen fibers in the tunicae media and in the adventitia. In the tunica media, foam cells appeared, and some smooth muscle cells showed vacuolated cytoplasm with dark pyknotic nuclei. Moreover, at the electron microscopic examination level, the nuclei in the endothelial cells seemed small and heterochromatic, and an apoptotic smooth muscle cell with a small heterochromatic nucleus and condensed cytoplasm appeared in the tunica media.

These light and electron microscopic findings confirm the role of endothelial dysfunction in the pathogenesis of FM-related disorders. The enhanced sympathetic activity could explain the FM-associated endothelial damage, and consequently, dysfunction caused by the chronic pain in FM. This chronically enhanced sympathetic overstimulation alters the vascular response to this chronic stimulation [30,31], which is reflected in the vascular wall structure, as observed in our study. Another finding in our research is increased intima-media thickness. This increase confirms the reported result, which states that “endothelial damage and dysfunction leads to endothelial mediated atherogenesis at the histological and radiological levels and depression, anxiety, and mood changes in FMS at the clinical level” [32]. This catecholamine-induced endothelial damage could be explained by the chronic activation of calcium channels, vascular wall membrane damage, persistent vascular spasms [33], and increased endothelin-1 secretion [34]. Another explanation was reported by Kohn et al. [35]. They mentioned that fibroblast deposition of collagen I and III in the arterial wall could result in medial fibrosis and intimal thickening and consequently increased intima-media thickness. They added that this collagen deposition is accompanied by vessel stiffening due to collagen fibers replacing vascular smooth muscle cells.

In FM patients, it was reported that there was reduced endothelial-dependent vasodilatation that was significantly correlated with the severity of pain parameters. The decrease in the vasodilator activity in FMS could be explained by the defective endothelial nitric oxide release [36,37,38] and decreased serum nitric oxide level [39]. These findings were confirmed in the current study, as there was a decrease in eNOS immunoreactivity, reduced expression at the mRNA level, and a decrease in the NO serum levels. The defective eNOS expression could impair the role of NO, as it has a vital role in vascular wall relaxation, blood flow regulation [40], and pain modulation [41]. As NO reduces fatigue through its stimulant effect on the mitochondrial biogenesis, especially in the skeletal muscles, and increases its performance due to the enhanced use of energy [42], FM patients who have reduced NO levels become more prone to muscle fatigue and pain [43].

In this study, the increased appearance of apoptotic cells in the form of small heterochromatic nuclei of endothelial cells and dark pyknotic nuclei of some smooth muscle cells was confirmed by the increased expression of caspase-3 along with a corresponding decrease in Bcl-2 at the mRNA level. The enhanced vascular wall apoptosis was previously reported by Dolcino et al. [44]. As detected in our study, the exaggerated apoptosis that is observed in fibromyalgia could be explained by fibromyalgia-associated oxidative stress [45]. The imbalance between oxidant and antioxidant production has a vital role in the pathogenesis of fibromyalgia-related disorders, increasing the severity of fibromyalgia manifestation [46] and consequently, the stimulation of endothelial and vascular wall apoptosis [47]. Additionally, the appearance of mast cells in the tunica adventitia was induced by oxidative stress. This finding coincided with that reported by Theoharides et al. [48]. They mentioned that systemic mastocytosis is commonly seen in patients who are affected by fibromyalgia.

In our study, the mitochondria showed destructed cristae with the appearance of autophagosomes when examined with an electron microscope, with enhanced expression of the autophagy markers LC 3 and BECN-1. The increased demonstrating the expression of the autophagy markers and the appearance of ultrastructure changes in the mitochondria, indicating marked autophagy of the mitochondria. It was reported that in early autophagosomes, the mitochondria are degraded in response to oxidative stress [46], making it a significant source of ROS, especially in conditions that are associated with mitochondrial damage and stress [49] such as in fibromyalgia [50]. The mitochondrial dysfunction in fibromyalgia agrees with the data published by Martínez-Lara et al., [9]. They stated that disturbed mitochondrial homeostasis and excessive mitochondrial autophagy are involved in the pathogenesis of fibromyalgia syndrome [9]. Excessive autophagy could be explained by cellular stresses such as endoplasmic reticulum stress, mitochondrial dysfunction, or oxidative stress, as in our study, all of which can cause induction of autophagy as a defense mechanism to eliminate the damaged cellular organelles [51].

This study’s microscopic examination of the cerebral cortex revealed nearly similar findings to the carotid arteries. This proves that both organs shared the same pathophysiological events in oxidative stress-enhanced apoptosis and excessive autophagy. These findings were also confirmed by the molecular expression of the apoptosis-related and autophagy-related genes. The light microscopic examination showed perineuronal empty spaces around shrunken pyramidal cells and vacuolation in the surrounding neuropil. These findings were also reported by Auer and Sutherland [52]. They documented the effect of hypoxia-induced oxidative stress on the cerebral cortex. They clarified that the vacuolation in the neuropil might be caused by the shrinkage of cells and the withdrawal of their processes due to cytoskeletal affection, resulting in pericellular spaces. Moreover, the ultrastructure examination of pyramidal and granular cells of the cerebral cortex revealed additional findings that were not detected in the carotid artery in the form of dilated rER cristae, which could indicate the involvement of endoplasmic reticulum stress in the pathogenesis of fibromyalgia-related cerebral pathological effects in addition to the previously discussed mitochondrial damage [52,53]. This ultrastructure finding was confirmed by higher expression of the CHOP gene at the mRNA level in the cerebral cortical tissue.

The endoplasmic reticulum (ER) is a cellular organelle that is highly sensitive to changes in intracellular homeostasis and extracellular stimuli, including oxidative stress [54], as well as diminished cerebral blood flow due to generalized endothelial and vascular dysfunction [55], as detected in our study. The ER is the main organelle that is responsible for protein synthesis and trafficking. It also regulates the autophagy pathways to maintain protein homeostasis in eukaryotic cells [56]. Meanwhile, the exposure of a cell to external or internal stressor stimuli such as chronic hypoxia, low-grade inflammation [57], and oxidative stress lead to the accumulation of misfolded proteins and cellular damage [58]. In addition, ER stress can enhance mitochondrial stress and stimulate the apoptosis of the cerebral cells [59]. Additionally, it has been reported that ER stress is directly proportional to endothelial damage, apoptosis, and dysfunction [60]. The coexistence of the ER stress pattern and that of the autophagosomes in the cerebral cortex cells could be explained by the stimulation of the autophagy process by the damaged endoplasmic reticulum to facilitate its partial engulfment in the autophagic vesicles to eliminate it [61].

The second main objective of the current study was to investigate the potential ameliorative effect of fisetin. The fisetin-treated group of animals showed improvement in the histological, biochemical, and molecular investigations. 

The administration of Fisetin as a therapeutic agent in the reserpine-induced fibromyalgia rat model was associated with significant improvement in the behavioral tests (Randall–Selitto paw pressure test, Hargreaves test, and tail suspension test), which indicated a marked improvement in the mechanic-hyperalgesia, thermal pain sensation periods of agitation, and immobility of rats with fibromyalgia. These findings confirm the results of Yao et al. [12]. Fisetin exerted an antioxidant effect, as there was a marked improvement in the oxidative stress markers in the fisetin-treated group, which was previously noticed by Maher et al. [11]. They stated that dietary polyphenols, including fisetin, can cross the blood–brain barrier, modulating intracellular antioxidant activity. As such, pretreatment and co-treatment with fisetin could attenuate the neurotoxicity in reserpine-induced fibromyalgia by inhibiting oxidative stress. Their results were confirmed by Rajendran and Ramachandran [62] and Yao et al. [12]. 

The antioxidant effect of fisetin is mediated by the inhibition of the enzymes that are involved in ROS generation, which stimulates gene expression in the enzymes that are responsible for ROS scavenging, the chelation of metal ions that is implicated in the formation of free radicals, and consequently, the motivation of antioxidant defenses [63]. Additionally, fisetin increases the availability of nitric oxide (NO). It prevents its oxidation, hence increasing its serum level [64], as detected in the current study, in the form of a high serum level of NO and increased eNOS immunoreactivity and its expression at the mRNA level.

Its antioxidant effect is proven by the apparent improvement in the markers of apoptosis that were observed in the carotid and cerebral tissues in the form of increased Bcl-2 and decreased caspase-3 gene expression. The anti-apoptotic effect of Fisetin can be explained by its inhibitory action of the intrinsic apoptotic pathway, which caused by hypoxia and oxidative stress, as it has an inhibitory effect known as the caspase cascade, which is considered to be the intrinsic pathway of apoptosis [65].

Moreover, in the current study, decreased gene expression in the autophagy markers (LC3 and BECN-1) was detected, but their expression was still significantly higher than it was the control groups. This finding indicated that fisetin has an autophagy-stimulating effect to help the organelles that have experienced reserpine-induced damage be degraded and expelled out of the fisetin-treated cell as part of the cellular defense system. The autophagy stimulating effect of fisetin has recently published by Sun et al. [66]. The mechanism by which fisetin induces autophagy was explained by its ability to induce autophagy in response to ER stress through the AMPK-independent pathway as well as its ability to up-regulate the expression of SIRT1 and to consequently activate SIRT1-mediated deacetylation, which leads to a diminished mTOR function with the net result induction of autophagy [67].

## 5. Conclusions

The results of the current study revealed that oxidative stress, enhanced apoptosis, and excessive autophagy are considered fundamental pathophysiologic mechanisms of reserpine-induced fibromyalgia in an experimental animal. Thus far, fisetin has been demonstrated to have an ameliorative effect on reserpine-induced fibromyalgia, as confirmed by an improvement in the behavioral tests, histological and ultrastructure studies of both cerebral and carotid tissue, and the biochemical and molecular markers of oxidative stress, apoptosis, and autophagy. One main conclusion is the intimate link between vascular pathology and cerebral pathology in fibromyalgia, which requires further investigation. 

## Figures and Tables

**Table 1:** Behavioral tests and serum oxidative stress parameters of all animal studied groups.

	Group I (-ve Control DMSO)	Group II (+ve Control Fisetin)	Group III (Reserpine Treated Group)	Group IV (Reserpine + Fisetin Treated Group)	F	*p*
Behavioral tests						
Randall-Selitto paw pressure test (grams)	220.40 ± 6.50	221.70 ± 6.34	83.90 ± 6.54 ^AB^	172.90 ± 5.45 ^ABC^	179.713	<0.001
Hargreaves test (seconds)	12.60 ± 3.50	13.90 ± 3.60	4.50 ± 1.58 ^AB^	11.30 ± 1.89 ^C^	23.836	<0.001
Tail suspension test (TST) (seconds)	7.20 ± 1.81	7.90 ± 2.28	48 ± 2.36 ^AB^	17.40 ± 4.50 ^ABC^	227.691	<0.001
Serum oxidative stress parameters						
MDA (μmol MDA/L)	14.41 ± 1.75	15.11 ± 1.26	36.08 ± 4.63 ^AB^	21.37 ± 3.54 ^ABC^	111.835	<0.001
TAC (mmol/L)	862.82 ± 138.28	873.45 ± 144.99	406.74 ± 56.76 ^AB^	815.39 ± 70 ^C^	39.942	<0.001
Serum NO (μmol/L)	24.82 ± 2.47	23.97 ± 2.78	13.83 ± 5.18 ^AB^	28.46 ± 8.85 ^C^	54.166	<0.001

Data are presented as mean ± SD. SD: standard deviation, F for ANOVA test. statistically significant if *p* ≤ 0.05. ^A^ comparison in relation to Group I (-ve control DMSO). ^B^ comparison in relation to Group II (+ve control Fisetin). ^C^ comparison in relation to Group III (Reserpine Treated group).

**Table 2:** Histological and molecular parameters of the carotid artery of all studied animal groups.

	Group I (-ve Control DMSO)	Group II (+ve Control Fisetin)	Group III (Reserpine Treated Group)	Group IV (Reserpine + Fisetin Treated Group)	F	*p*
IMT	32.94 ± 6.53	33.07 ± 5.09	44.02 ± 11.94 ^AB^	36.03 ± 7.76 ^C^	5.979	0.001
Area % of collagen	8.92 ± 2.20	8.34 ± 1.83	14.30 ± 3.59 ^AB^	9.38 ± 3.72 ^C^	12.915	<0.001
Total number of mitochondria	6.07 ± 1.33	5.47 ± 0.99	14.87 ± 1.30 ^AB^	6.46 ± 1.06 ^BC^	22.620	<0.001
Area % of eNOS	2.41 ± 0.13	2.13 ± 0.15	1.63 ± 0.75 ^AB^	2.11 ± 0.08 ^C^	13.549	<0.001
eNOS gene expression (2^−∆∆CT^)	1 ± 0.02	1.02 ± 0.03	0.47 ± 0.11 ^AB^	0.90 ± 0.13 ^BC^	84.120	<0.001
Bcl2 gene expression (2^−∆∆CT^)	1 ± 0.03	1.04 ± 0.03	0.60 ± 0.18 ^AB^	1.19 ± 0.19 ^AC^	37.860	<0.001
Caspase-3 gene expression (2^−∆∆CT^)	1 ± 0.02	1.02 ± 0.06	2.33 ± 0.36 ^AB^	1.41 ± 0.19 ^ABC^	91.075	<0.001
LC-3 gene expression (2^−∆∆CT^)	1 ± 0.03	1.01 ± 0.02	2.28 ± 0.22 ^AB^	1.60 ± 0.21 ^ABC^	152.775	<0.001
BECN-1 gene expression (2^−∆∆CT^)	1.01 ± 0.02	1.03 ± 0.02	2.24 ± 0.29 ^AB^	1.46 ± 0.17 ^ABC^	116.248	<0.001
CHOP gene expression (2^−∆∆CT^)	1.05 ± 0.07	1.02 ± 0.04	1.36 ± 0.17 ^AB^	1.21 ± 0.12 ^BC^	15.130	<0.001
TNF-α gene expression (2^−∆∆CT^)	1.01 ± 0.03	1.02 ± 0.04	2.28 ± 0.19 ^AB^	1.47 ± 0.29 ^ABC^	201.37	<0.001

Data are presented as mean ± SD. SD: standard deviation, F for ANOVA test. statistically significant if *p* ≤0.05. ^A^ comparison in relation to Group I (-ve control DMSO). ^B^ comparison in relation to Group II (+ve control Fisetin). ^C^ comparison in relation to Group III (reserpine-treated group).

**Table 3:** Histological and molecular parameters of the cerebral cortex of all studied animal groups.

	Group I (-ve Control DMSO)	Group II (+ve Control Fisetin)	Group III (Reserpine Treated Group)	Group IV (Reserpine + Fisetin Treated Group)	F	*p*
Total number of mitochondria	9.20 ± 1.37	10.07 ± 0.88	21.07 ± 1.33	11 ± 1.19	48.419	<0.001
Area % of eNOS	1.90 ± 0.18	1.81 ± 0.17	0.92 ± 0.33 ^AB^	1.55 ± 0.28 ^ABC^	43.306	<0.001
eNOS gene expression (2^−∆∆CT^)	1 ± 0.02	1.04 ± 0.03	0.57 ± 0.14 ^AB^	0.91 ± 0.13 ^BC^	49.354	<0.001
Bcl2 gene expression (2^−∆∆CT^)	1.01 ± 0.03	1.04 ± 0.03	0.62 ± 0.16 ^AB^	1.12 ± 0.13 ^C^	42.636	<0.001
Caspase-3 gene expression (2^−∆∆CT^)	1.01 ± 0.02	1.03 ± 0.03	2.36 ± 0.24 ^AB^	1.52 ± 0.14 ^ABC^	209.567	<0.001
LC-3 gene expression (2^−∆∆CT^)	1 ± 0.02	1.01 ± 0.02	1.74 ± 0.23 ^AB^	1.18 ± 0.11 ^ABC^	73.270	<0.001
BECN-1 gene expression (2^−∆∆CT^)	1.01 ± 0.02	1.01 ± 0.02	2.39 ± 0.23 ^AB^	1.44 ± 0.14 ^ABC^	222.45	<0.001
CHOP gene expression (2^−∆∆CT^)	1.01 ± 0.07	1.03 ± 0.04	2.03 ± 0.21 ^AB^	1.20 ± 0.19 ^BC^	24.611	<0.001
TNF-α gene expression (2^−∆∆CT^)	1.01 ± 0.03	1.04 ± 0.02	2.18 ± 0.19 ^AB^	1.33 ± 0.19 ^ABC^	213.39	<0.001

Data are presented as mean ± SD. SD: standard deviation, F for ANOVA test. statistically significant if *p* ≤ 0.05. ^A^ comparison in relation to Group I (-ve control DMSO). ^B^ comparison in relation to Group II (+ve control fisetin). ^C^ comparison in relation to Group III (reserpine-treated group).

## Data Availability

Not applicable.
